# Multiparametric quantitative structural and functional cardiac MRI in orthotopic heart transplant recipients with cardiac allograft vasculopathy

**DOI:** 10.1007/s10554-025-03384-z

**Published:** 2025-04-17

**Authors:** Sandra Quinn, Roberto Sarnari, Andrew Zbihley, Daniel Sherlock, Connor Raikar, Joshua Engel, Havisha Pedamallu, Kai Lin, Kambiz Ghafourian, Daniel C. Lee, Esther E. Vorovich, Clyde W. Yancy, Vera H. Rigolin, Jon W. Lomasney, James C. Carr, Bradley D. Allen, Michael Markl

**Affiliations:** 1 Department of Radiology, Northwestern University Feinberg School of Medicine, 737 N Michigan Ave, Suite 1600, Chicago, IL 60611 USA; 2 Division of Cardiology, Department of Medicine, Northwestern University, Chicago, IL USA; 3 Department of Pathology, Northwestern University Feinberg School of Medicine, Chicago, IL USA; 4 Department of Biomedical Engineering, McCormick School of Engineering, Northwestern University, Chicago, IL USA

**Keywords:** Heart transplant, Cardiac allograft vasculopathy, ECV, T2, Feature-tracking strain

## Abstract

The aim of this study was to verify if multiparametric quantitative CMR can detect mild-to-moderate cardiac allograft vasculopathy (CAV) in patients post-orthotopic heart transplant (OHT). 51 patients (age = 50.0 ± 13.6 years, 29% female) post-OHT 0–6 years (mean 3.2 ± 1.5 years) who underwent CMR from 2011 to 2019 were retrospectively included. Multiparametric CMR included CINE imaging covering the left ventricle (LV), pre- and post-contrast T1 mapping, and T2 mapping, extracellular volume fraction (ECV) calculation, and 2D-feature tracking strain. CAV0 (‘CAV negative’) patient variables were compared with CAV1-CAV2 (‘CAV positive’) variables. Logistic regression was used to determine predictors of CAV status. Myocardial T2 was higher in CAV positive compared with CAV negative patients (54.5 ± 7.7 ms vs. 50.2 ± 3.3 ms, *p* < 0.05), as was ECV (31.3 ± 5.3% vs. 27.4 ± 4.1%, *p* < 0.05). Radial and circumferential peak systolic strain rates were attenuated in CAV positive vs. CAV negative patients (radial: 1.4 ± 0.4 s-1 vs. 1.8 ± 0.3 s-1, circumferential: -0.9 ± 0.2 s-1 vs. -1.1 ± 0.1 s-1, *p* < 0.05),as well as circumferential and longitudinal peak diastolic strain rates (0.7 ± 0.7 s-1 vs. 1.0 ± 0.5 s-1, and 0.8 ± 0.3 s-1 vs. 0.9 ± 0.3 s-1, *p* < 0.05, respectively). CAV positive vs. negative status correlated with ECV (rho 0.41, *P* < 0.01), T2 (rho 0.29, *p* < 0.05), radial and circumferential peak systolic strain rate (rho − 0.48, *P* < 0.01 and rho 0.47, *p* < 0.001, respectively), and circumferential and longitudinal peak diastolic strain rates (rho − 0.34, *p* < 0.05 and rho − 0.35, *p* < 0.01, respectively). Logistic regression revealed that a model including ECV, peak radial and circumferential systolic strain rates and longitudinal diastolic strain rate was significant for distinguishing CAV positive vs. negative status with a receiver operator characteristic area under curve of 0.85 ± 0.06 (CI 0.73–0.97), *p* < 0.005. A model combining functional (strain) and tissue parameters (ECV) was predictive of CAV status, indicating the potential of multiparametric CMR for non-invasive prediction of CAV status in OHT recipients.

## Introduction

Orthotopic heart transplant (OHT) is an established treatment for end-stage heart failure which improves long-term mortality in recipients [1, 2]. Despite ongoing advances in clinical management of the post-OHT patient, cardiac allograft vasculopathy (CAV) remains a major long-term complication, and it continues to be one of the leading causes of mortality in these patients long-term [2, 3]. CAV is characterized as a form of chronic rejection, with development of accelerated inflammatory fibroproliferative disease in either epicardial coronary arteries, intramural microvasculature, or both. The incidence of CAV increases in OHT recipients over time, with 8% of recipients developing CAV within the first year post OHT, and over 45% developing CAV by 10 years [2]. As CAV progresses over time, the myocardium undergoes altered perfusion, chronic ischemic injury, and ultimately allograft failure [4]. Early and ongoing screening and detection of CAV is thus paramount, as medical treatments can be implemented to slow progression of CAV [5–7].

Invasive coronary angiography (ICA) has a Class I recommendation (level of evidence C) from the 2010 International Society of Heart and Lung Transplantation (ISHLT) guidelines for serial CAV surveillance in the heart transplant recipient and is recommended to occur annually or bi-annually [8]. ICA findings, combined with right heart catheterization (RHC) and transthoracic echocardiographic (TTE) parameters, are used as the ‘gold standard’ for CAV surveillance and grading [8]. CAV grading ranges from CAV_0_ to CAV_3_ (non-significant, mild, moderate or severe disease, respectively) as per current ISHLT classification [9].

ICA is associated with a low yet significant risk of major complications including stroke, myocardial infarction, and death [10] as well as cumulative radiation exposure from repeated ICA procedures, and therefore there is an unmet need for an alternative non-invasive test to determine the presence or absence of CAV.

Cardiac MRI (CMR) has potential for non-invasive detection of CAV without the risks associated with repeat invasive left catheterization procedures. The aim of this study therefore was to evaluate the diagnostic value of cardiac structure-function CMR measures including global cardiac left ventricular (LV) and right ventricular (RV) function, Native T1, extracellular volume (ECV), T2, and two-dimensional (2D) feature-tracking strain (FTS) for non-invasive assessment of CAV severity.

## Methods

### Study cohort

A database of OHT patients who underwent comprehensive CMR at a single tertiary centre was queried. Inclusion criteria were: ≥ 18 years of age, CMR 0–6 years post-OHT, and a clinically documented ICA, RHC and TTE for formulation of ISHLT CAV grade at the time of CMR [8]. Exclusion criteria included any patients with active significant allograft rejection i.e., >2R acute cardiac allograft rejection (ACAR) or any active humoral rejection at the time of CMR. Institutional research board approval was obtained for this study. Informed consent was obtained from participants who underwent prospective recruitment, whereas some subjects received clinical CMR and were enrolled retrospectively with waiver of consent.

### CMR acquisition

CMR exams were performed on a 1.5-T MR system (Magnetom Aera or Avanto, Siemens, Erlangen, Germany), including assessment of cardiac function and tissue parameter by 2D balanced steady-state free precession (bSSFP) cine imaging, pre- and post-gadolinium T1 mapping, and T2 mapping.

The complete heart was imaged using cine-bSSFP in both short-axis (8–12 slices per stack) and long-axis (2-chamber, 3-chamber, and 4-chamber) views. The short-axis images included both ventricles from the base to the apex and were reconstructed into 25 cardiac time frames. The imaging parameters used were a repetition time (TR) of 2.6-3.0 ms, an echo time (TE) of 1.1–1.3 ms, a flip angle of 50–87 degrees, a bandwidth/pixel of 930 Hz, a generalized auto-calibrating partially parallel acquisitions acceleration (GRAPPA) with acceleration factor *R* = 2, an in-plane resolution of 1.5–2.3 mm², and a slice thickness of 6–8 mm.

For native and post-contrast T1 and T2 mapping, breath-holding was used to acquire images at three identical short-axis locations at the base, mid-ventricle, and apex. T1 mapping was achieved using single-shot modified Look Locker Inversion Recovery (MOLLI) native images (pre-gadolinium) and post-gadolinium images acquired 15 min after contrast injection (Gadavist or Magnevist, 0.1 mmol/kg, Bayer, Leverkusen, Germany) with MOLLI sequences occurring in a 5(3)3 pattern over 11 heartbeats [11]. The imaging parameters were as follows: TE/TR = 1.0-1.3ms/2.5-4.2ms, spatial resolution of 1.0–2.1 mm x 1.5–2.5 mm, slice thickness of 8 mm, and a flip angle of 35 degrees. Motion correction was applied to the MOLLI images with different inversion times, and parametric LV T1-maps were calculated.

T2 mapping was based on the successive acquisition of three T2-prepared SSFP images with varying T2 preparation times (0, 24, 55 ms). The imaging parameters used for T2 mapping were a TE of 1.1–1.4 ms, a TR of 2.2–2.6 ms, a spatial resolution of 1.5–2.1 mm x 2.0–2.5 mm, a slice thickness of 8 mm, a diastolic acquisition window of 270 ms, and a flip angle of 70 degrees [12].

### CMR post-processing

Commercial software (CVI42, Circle Cardiovascular Imaging, V5.13) was used to calculate measures of biventricular function from cine bSSFP images obtained in both short and long-axis views. Manual contouring was performed to segment the endocardium and epicardium of the LV and RV, with trabeculae and papillary muscles excluded from the analysis. Subsequently volumetric and functional parameters were determined, specifically LV and RV end diastolic volume (ml), end systolic volume (ml), stroke volume (ml), cardiac output (L/min), ejection fraction (%) and LV myocardial mass (g).

Global native T1 and T2 values were calculated based on the average of all segmental LV values as per 16-segment American Heart Association model. Native and post-contrast blood pool were contoured manually. ECV was calculated as: ECV=(Δ[1/T1myocardium]/Δ[1/T1bloodpool]x[1 -haematocrit]).

LV 2D-FTS was also applied. 2D global radial, circumferential and longitudinal peak strain and systolic and diastolic strain rates were evaluated with FTS post-processing software (CVI42, Circle Cardiovascular Imaging, V5.13).

### CAV assessment

ICA images were retrospectively assessed in conjunction with RHC, TTE and clinical data. CAV were graded from CAV_0_ (non-significant/non-detectable), CAV_1_ (mild), CAV_2_ (moderate) or CAV_3_ (severe) as per ISHLT criteria [8, 9] as the clinical reference standard for CAV severity.

### Statistical analysis

Descriptive statistics for continuous variables are presented as mean ± SD, and categorical variables as counts with percentage. Repeat measures were compared with independent t-test (parametric) or Mann-Whitney U Test (non-parametric). Correlation coefficient analyses were performed with Pearson or Spearman’s test. Receiver operator characteristic (ROC) curves with area under curve (AUC) values with 95% confidence intervals (CI) were determined for individual CMR parameters and multiparametric CMR using binary logistic regression. *P* < 0.05 was considered statistically significant.

## Results

### Patient characteristics

In total, 51 patients received a CMR within the first 5 years post-OHT, with a mean age of 50.0 ± 13.6 years, 29% female. The mean time from OHT to CMR was 3.2 ± 1.5 years, and ICA was performed within a mean time of 0.7 ± 0.5 years from CMR. Of this group, 26 patients (51%) had no CAV as per ISHLT criteria i.e. the “CAV negative” group, and 25 patients (49%) had a diagnosis of CAV i.e. the “CAV positive” group. Of the CAV positive group, 23 patients had mild CAV (CAV_1_) and 2 patients had moderate CAV (CAV_2_), with a mean CAV grade of 1.2 ± 0.1. No patients had severe CAV (CAV_3_).

Comparing CAV negative and CAV positive groups, there was no significant difference in LV ejection fraction (52.9 ± 11.1% vs. 48.4 ± 10.9%), stroke volume (59.9 ± 18.8 ml vs. 59.8 ± 21.5 ml) or end diastolic volume (115.1 ± 32.8 ml ± 123.7 ± 33.7 ml). Systolic blood pressure was significantly higher in the CAV positive group (116.8 ± 16.0 mmHg vs. 126.8 ± 15.1 mmHg, *p* < 0.05) (Table 1).

**Table 1:** Subject characteristics for (1) OHT patients without CAV (‘CAV negative’) (2) with mild to moderate CAV (‘CAV positive’) and (3) total patients included in this study. ACAR = acute cardiac allograft ejection; cav = cardiac allograft vasculopathy; cmr = cardiac MRI; ica = invasive coronary angiogram; ishlt = international society of heart and lung transplant; lv = left ventricle; oht = orthotopic heart transplant rv = right ventricle

Patient Data	CAV negative	CAV positive	Total	*P*-value
Patient demographics				
Number of patients (n)	26	25	51	N/A
Gender (n, %)				
Male	17 (65%)	19 (76%)	36 (71%)	0.41
Female	9 (35%)	6 (24%)	15 (29%)	
Age at time of CMR (years)	49.2 ± 13.2	50.9 ± 14.3	50.0 ± 13.6	0.49
Time from OHT to CMR (years)	3.3 ± 1.7	3.1 ± 1.4	3.2 ± 1.5	0.55
Time from CMR to ICA (years)	0.8 ± 0.6	0.6 ± 0.4	0.7 ± 0.5	0.13
Height (cm)	174.0 ± 9.0	174.4 ± 9.8	174.2 ± 9.2	0.87
Weight (kg)	82.1 ± 14.6	89.9 ± 16.6	85.9 ± 16.0	0.08
Body Mass Index (kg/m^2^)	27.2 ± 5.2	29.6 ± 5.1	28.4 ± 5.3	0.11
Body Surface Area (m^2^)	2.1 ± 0.2	2.0 ± 0.2	2.0 ± 0.2	0.08
Systolic Blood Pressure (mmHg)	116.8 ± 16.0	126.8 ± 15.1	121.8 ± 16.3	**< 0.05**
Diastolic Blood pressure (mmHg)	75.2 ± 10.3	75.7 ± 11.8	75.4 ± 10.9	0.87
Heart rate (beats per minute)	89.7 ± 10.4	92.0 ± 13.5	90.6 ± 12.0	0.42
**CMR Volumetrics and Function**				
LV end diastolic volume (ml)	115.1 ± 32.8	123.7 ± 33.2	119.3 ± 13.9	0.24
LV end systolic volume (ml)	55.2 ± 25.5	63.9 ± 23.4	59.5 ± 24.6	0.21
LV stroke volume (ml)	59.9 ± 18.8	59.8 ± 21.5	59.9 ± 20.0	0.99
LV cardiac output (L/min)	5.3 ± 1.5	5.3 ± 1.6	5.3 ± 1.6	0.92
LV ejection fraction (%)	52.9 ± 11.1	48.4 ± 10.9	50.7 ± 11.1	0.07
Myocardial mass diastole (g)	95.2 ± 22.1	103.4 ± 20.6	99.2 ± 21.6	0.18
RV end diastolic volume (ml)	128.3 ± 34.3	140.9 ± 37.8	134.5 ± 36.2	0.09
RV end systolic volume (ml)	74.6 ± 30.0	86.0 ± 32.6	80.2 ± 30.6	0.10
RV stroke volume (ml)	53.8 ± 19.7	54.9 ± 23.8	54.3 ± 21.6	0.86
RV cardiac output (l/min)	4.7 ± 1.8	4.8 ± 1.9	4.8 ± 1.8	0.83
RV ejection fraction (%)	42.3 ± 12.0	39.4 ± 14.2	40.9 ± 13.1	0.43
**ISHLT CAV severity**				
Mean CAV grade	0.0 ± 0.0	1.2 ± 0.1	0.6 ± 0.7	**< 0.001**
0 (non-significant) (n, %)	26 (100%)	0 (0%)	26 (51%)	**< 0.05**
1 (mild) (n, %)	0 (0%)	23 (92%)	23 (45%)	
2 (moderate) (n, %)	0 (0%)	2 (8%)	2 (4%)	
3 (severe) (n, %)	0 (0%)	0 (0%)	0 (0%)	
**Rejection history**				
ACAR (*≥ 2*R)				0.14
No	23 (88%)	18 (72%)	41 (80%)	
Yes	3 (12%)	7 (28%)	10 (20%)	
Humoral allograft rejection				0.67
No	23 (88%)	23 (92%)	46 (90%)	
Yes	3 (12%)	2 (8%)	5 (10%)	

### Tissue and strain characteristics for CAV negative and positive groups

ECV was significantly higher in the CAV positive group when compared to the CAV negative group (31.3 ± 5.3% vs. 27.4 ± 4.1% *p* < 0.05), as was LV T2 (54.5 ± 7.7 ms vs. 50.2 ± 3.3 ms, *p* < 0.05). There were no significant differences for native T1 (Table 2).

**Table 2:** Comparison of tissue characterization and feature tracking strain characteristics for (1) OHT patients without CAV (‘CAV negative’) (2) with mild to moderate CAV (‘CAV positive’) and (3) total patients included in this study. N/A = not applicable

CMR Evaluation		CAV negative	CAV positive	Total	*P*-value
Tissue Characterization					
Native T1 mapping (ms)		1005.2 ± 47.3	1033.0 ± 57.7	1018.8 ± 54.0	0.07
Post Gadolinium T1 mapping (ms)		426.5 ± 53.5	405.8 ± 89.4	416.4 ± 73.3	0.33
Extracellular Volume (%)		27.4 ± 4.1	31.3 ± 5.3	29.3 ± 5.1	**< 0.05**
T2 mapping (ms)		50.2 ± 3.3	54.5 ± 7.7	52.3 ± 6.2	**< 0.05**
**2D Feature Tracking Strain**					
Number of patients (n)		22	18	40	N/A
Peak systolic strain %	Radial	28.6 ± 7.7	24.0 ± 6.7	26.5 ± 7.5	0.05
	Circumferential	-17.2 ± 3.0	-15.4 ± 3.4	-16.4 ± 3.3	0.16
	Longitudinal	-13.5 ± 2.6	-11.3 ± 3.9	-12.7 ± 3.3	0.08
Global peak systolic strain rate (s^− 1^)	Radial	1.8 ± 0.3	1.4 ± 0.4	1.6 ± 0.4	**< 0.005**
	Circumferential	-1.1 ± 0.1	-0.9 ± 0.2	-1.0 ± 0.2	**< 0.005**
	Longitudinal	-0.8 ± 0.1	-0.7 ± 0.2	-0.8 ± 0.2	0.13
Global peak diastolic strain rate (s^− 1^)	Radial	-0.9 ± 1.8	-0.5 ± 1.7	-0.7 ± 1.8	0.25
	Circumferential	1.0 ± 0.5	0.7 ± 0.7	0.9 ± 0.6	**< 0.05**
	Longitudinal	0.9 ± 0.3	0.8 ± 0.3	0.8 ± 0.3	**< 0.05**

Of the 51 patients included, 40 of the patient CMRs were suitable for 2D-FTS post processing, with 11 excluded due to suboptimal cine quality for FTS purposes. In these patients, the global radial peak systolic strain rate was significantly attenuated in the CAV positive group when compared to CAV negative group (1.4 ± 0.4s^− 1^ vs. 1.8 ± 0.3 s^− 1^, *p* < 0.005) as was the global circumferential peak systolic strain rate (-0.9 ± 0.2 s^− 1^ vs. -1.1 ± 0.1 s^− 1^ vs., *p* < 0.005). The global circumferential peak diastolic strain rate was significantly attenuated in the CAV positive group when compared to CAV negative group (0.7 ± 0.7 s^− 1^ vs. 1.0 ± 0.5 s^− 1^*p* < 0.05) as was the global longitudinal peak diastolic strain rate (0.8 ± 0.3 s^− 1^ vs. 0.9 ± 0.3 s^− 1^, *p* < 0.005).

### Correlation of CAV status with tissue and strain characteristics

There were significant correlations between CAV positive or negative status and ECV (*rho* 0.41; *p* < 0.01) and myocardial T2 (*rho* 0.29, *p* < 0.05). In addition, there were significant associations for CAV negative or positive status with attenuated strain parameters: radial peak systolic strain rate (*rho* − 0.48, *p* < 0.005); circumferential peak systolic strain rate (*rho* 0.47, *p* < 0.001), circumferential peak diastolic strain rate (*rho* − 0.34, *p* < 0.005) and longitudinal peak diastolic strain rate (*rho* − 0.35, *p* < 0.05) (Table 3).

**Table 3:** Correlation of tissue characterization and feature tracking strain characteristics for (1) OHT patients CAV_0_ (‘CAV negative’) vs. (2) OHT patients CAV_1_ to CAV_2_ (‘CAV positive’)

CMR tissue characteristics and strain parameters		rho coefficient	*P* value
Native T1 mapping (ms)		0.25	0.06
Post-Gadolinium T1 mapping (ms)		-0.19	0.16
Extracellular volume fraction (%)		0.41	**< 0.01**
T2 mapping (ms)		0.29	**< 0.05**
Peak systolic strain (%)	Radial	-0.25	0.12
	Circumferential	0.23	0.16
	Longitudinal	0.25	0.12
Systolic strain rate (s^− 1^)	Radial	-0.48	**< 0.005**
	Circumferential	0.47	**< 0.001**
	Longitudinal	0.25	0.12
Diastolic strain rate (s^− 1^)	Radial	0.19	0.25
	Circumferential	-0.34	**< 0.05**
	Longitudinal	-0.35	**< 0.05**

### Correlations between tissue characteristics and strain parameters

**Fig. 1:**
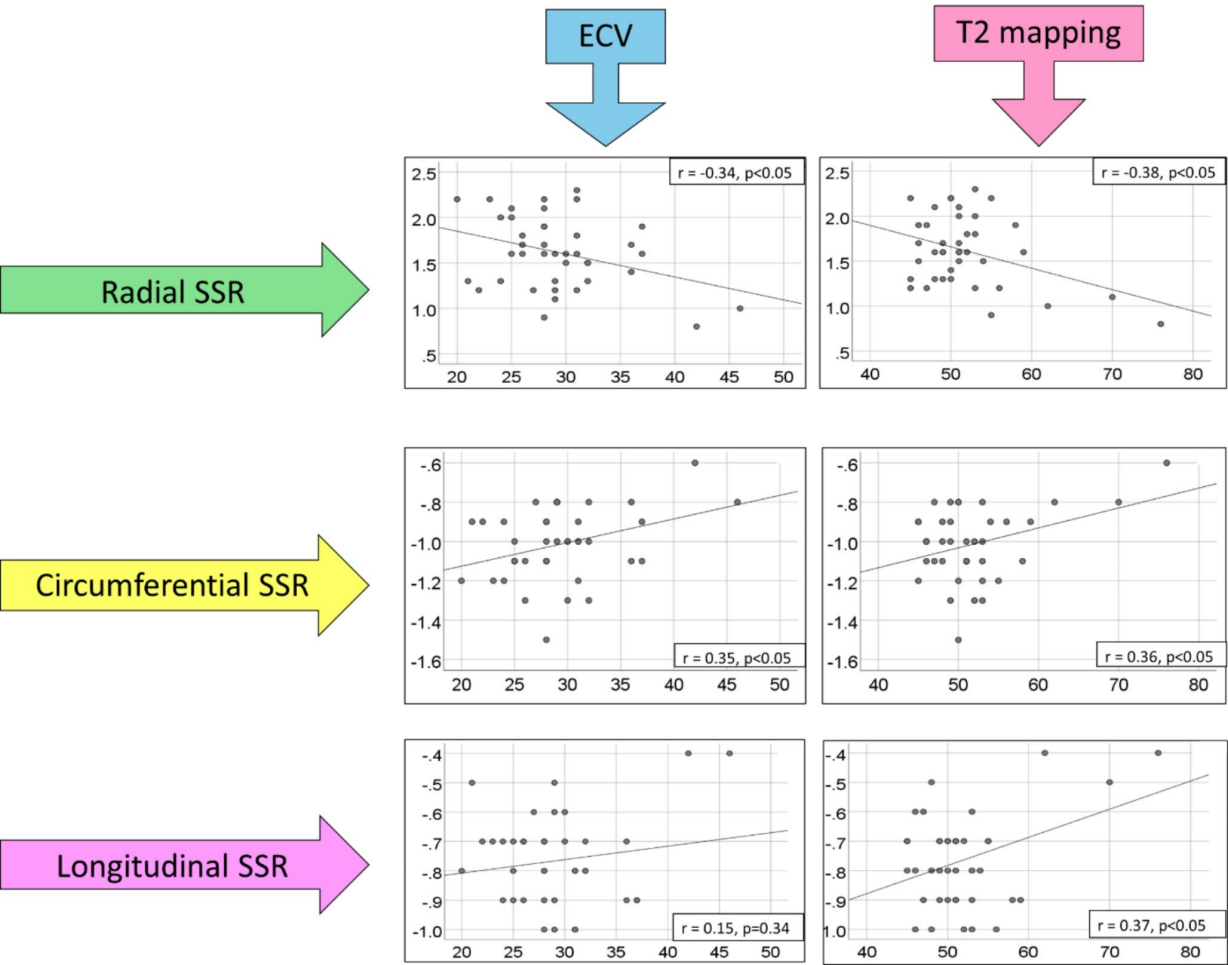
Correlations between myocardial systolic strain rates (SSR) and extracellular volume (ECV) and T2 mapping

In this patient group overall, there were significant structure-function relationships between elevated ECV and reduced radial peak systolic strain rate (*r* -0.34, *p* < 0.05), and depressed circumferential peak systolic strain rate (*r* 0.35, *p* < 0.001). Elevated LV T2 was associated with reduced peak systolic strain rate in all dimensions: radial (*r* -0.38, *p* < 0.05), circumferential (*r* 0.36, *p* < 0.05) and longitudinal (*r* 0.37, *p* < 0.05) (Fig. 1).

### Individual CMR parameters for CAV detection

ROC curves for ECV demonstrated good discrimination between CAV negative and CAV positive (AUC 0.74 ± 0.07, 95% CI 0.60–0.88, *p* < 0.05). Global peak systolic strain rates in the radial and circumferential dimensions could also discriminate CAV negative vs. positive status: radial: AUC 0.77 ± 0.08, 95% CI 0.63–0.92, *p* < 0.005; circumferential AUC 0.77 ± 0.08, 95% CI 0.61–0.93, *p* < 0.005. In addition, global longitudinal peak diastolic strain rate also demonstrated good discrimination (AUC 0.70 ± 0.09, CI 0.53–0.87, *p* < 0.05) (Table 4).

**Table 4:** Receiver operator characteristic (ROC) area under curve (AUC) analysis with 95% confidence interval (CI) for discrimination of CAV negative vs. positive status using individual and multiparametric quantitative CMR tissue and feature tracking strain characteristics

ROC parameter		AUC (mean ± SE)	95% CI	*P* value
Tissue Characteristics				
Native T1 (ms)		0.66 ± 0.08	0.50–0.81	0.06
Post-Gadolinium T1 (ms)		0.62 ± 0.08	0.46–0.77	0.16
ECV (%)		0.74 ± 0.07	0.60–0.88	**< 0.05**
T2 (ms)		0.66 ± 0.08	0.51–0.81	**< 0.05**
**Strain**				
Peak systolic strain (%)	Radial	0.64 ± 0.09	0.47–0.82	0.12
	Circumferential	0.63 ± 0.09	0.46–0.81	0.15
	Longitudinal	0.64 ± 0.09	0.46–0.83	0.12
Global peak systolic strain rate (s^− 1^)	Radial	0.77 ± 0.08	0.63–0.92	**< 0.005**
	Circumferential	0.77 ± 0.08	0.61–0.93	**< 0.005**
	Longitudinal	0.64 ± 0.09	0.47–0.82	0.13
Global peak diastolic strain rate (s^− 1^)	Radial	0.61 ± 0.09	0.43–0.79	0.24
	Circumferential	0.69 ± 0.09	0.52–0.86	**< 0.05**
	Longitudinal	0.70 ± 0.09	0.53–0.87	**< 0.05**
**Multiparametric (AUC ≥ 0.70)**				
ECV + Global peak systolic strain rate (circumferential)		0.80 ± 0.08	0.66–0.95	**< 0.005**
ECV + Global peak systolic strain rate (radial)		0.84 ± 0.07	0.71–0.97	**< 0.005**
ECV + Global peak systolic strain rates (radial and circumferential)		0.83 ± 0.06	0.71–0.96	**< 0.005**
ECV + Global peak diastolic strain rate (longitudinal)		0.80 ± 0.08	0.65–0.95	**< 0.005**
Global peak systolic strain rates (radial and circumferential), + Global peak diastolic strain rate (longitudinal)		0.77 ± 0.07	0.63–0.92	**< 0.005**
ECV + Global peak systolic strain rates (radial and circumferential), + Global peak diastolic strain rate (longitudinal)		0.85 ± 0.06	0.73–0.97	**< 0.005**

### Multiparametric CMR for CAV detection

**Fig. 2:**
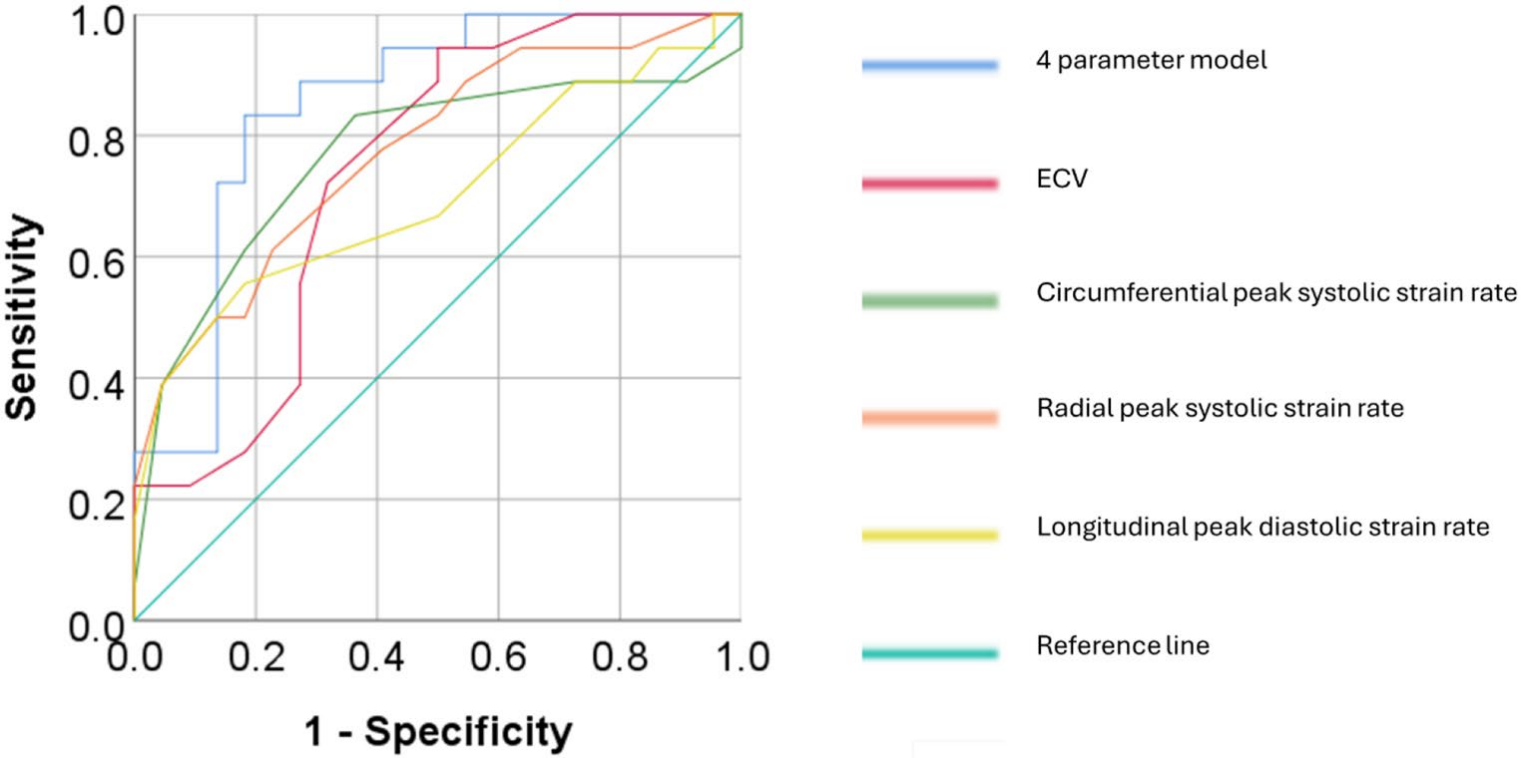
Receiver operator characteristic curves for extracellular volume (ECV), radial and circumferential systolic strain rates and longitudinal diastolic strain rates, and all 4 parameters combined

Binary logistic regression was performed to assess the impact of a set of predictor variables with an AUC ≥ 0.70 and *p* < 0.05 for determining CAV negative or positive status. The model contained 4 independent variables (ECV, global radial peak systolic strain rate, global circumferential peak systolic strain rate, and global longitudinal peak diastolic strain rate). The full model was statistically significant χ2 = 17.9, *p* < 0.005, indicating that the model was able to distinguish between patients who were CAV negative vs. CAV positive. The model as a whole correctly classified 80% of cases, with a ROC AUC of 0.85 ± 0.06, 95% CI 0.73–0.97, *p* < 0.005 (Fig. 2), with a sensitivity of 86%, specificity of 72%, negative predictive value of 79% and positive predictive value of 81%.

## Discussion

**Fig. 3:**
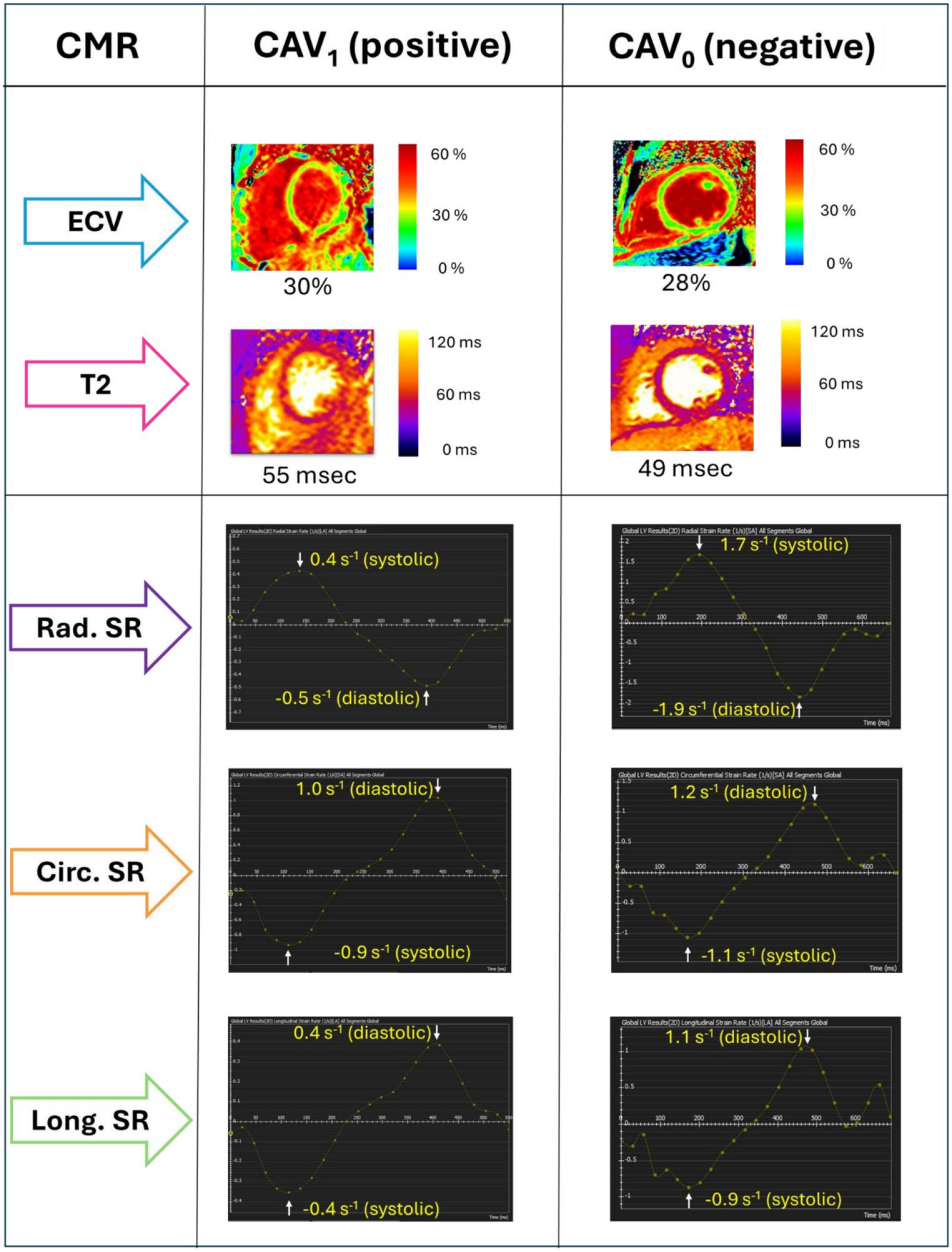
Representative cases of a CAV_1_ (CAV positive) and CAV_0_ (CAV negative) OHT recipients; both patients are 4 years post-OHT and have an LVEF of 55–60%, and no history of clinically significant allograft rejection. The CAV positive patient has higher ECV and T2 values than the CAV negative patient, with reduced systolic and diastolic strain rates. CAV = cardiac allograft vasculopathy; ECV = extracellular volume; Rad. = radial; Circ. = circumferential; Long. = longitudinal; SR = strain rate

Our study demonstrates the potential of CMR based tissue and function assessment for the non-invasive identification of CAV severity in OHT patients. Our main findings include: (1) myocardial ECV and T2 were elevated in OHT patients with mild to moderate CAV, (2) presence of CAV was associated with attenuated radial and circumferential systolic strain rates, and (3) attenuated circumferential and longitudinal diastolic strain rates (Fig. 3). Furthermore, significant correlations between tissue characteristics and strain parameters were detected, suggesting a relationship between the myocardial tissue changes that occur in CAV and subclinical myocardial dysfunction, as detected by strain parameters. A logistic regression model combining ECV with strain parameters demonstrated good discrimination for mild to moderate CAV detection. Overall, this study suggests that multiparametric quantitative CMR, combining tissue characterization and strain parameters, may be beneficial in non-invasive detection of CAV.

Previous studies have demonstrated the potential of CMR for detection of CAV severity. Abbasi et al. (2022) demonstrated that when compared to control patients, OHT patients with ISHLT severity grades of CAV_2_-CAV_3_ had greater MOLLI native T1, global and peak ECV and global and peak T2, with global T2 significantly elevated in CAV_2_-CAV_3_ patients when compared to CAV_1_ patients [13]. In a paediatric retrospective study with a population with low incidence of rejection or high-grade CAV, increased native T1, ECV and T2 were associated with CAV history with significant differences between patients with ISHLT CAV_0_ when compared to CAV_1_ [14]. The results of our study support these findings that T2 and ECV may be beneficial in the detection of CAV.

Strain assessment by different CMR techniques, has demonstrated its potential in CAV assessment. Erbel et al. (2016), by using strain-encoded (SENC) CMR, demonstrated that CMR early diastolic strain rate was independently associated with microvascular disease and clinical outcomes in patients with CAV [15]. In addition, early diastolic strain rate was independently associated with epicardial coronary disease in 2 previous studies, by using SENC and myocardial tagging for strain calculation [16, 17]. In our study, FTS was utilized for strain calculation: due to FTS high reproducibility^18^ and low time-consuming post-processing, FTS is the most widely utilized post-processing technique in clinical practice. We demonstrated attenuated radial and circumferential systolic strain rates and circumferential and longitudinal diastolic strain rates in OHT patients with mild to moderate CAV when compared to OHT patients without CAV. These attenuated parameters suggest subclinical myocardial dysfunction that occurs in patients with mild to moderate CAV, involving as the diastolic and the systolic phase, that is not detected with macroscopic functional parameters as LV ejection fraction and LV stroke volume. Interestingly, ECV correlated significantly with radial and circumferential systolic strain rate, and T2 correlated significantly with systolic strain rate in all dimensions. These findings suggest that myocardial tissue changes that occur in mild to moderate CAV, as detected by increased ECV and T2 mapping, may result in subclinical dysfunction of the myocardium as detected by feature tracking-calculated strain parameters.

In addition to procedure related risks, the diagnostic accuracy of ICA for CAV detection can also be limited and with suboptimal sensitivity in some cases [16, 19, 20], for example to the diffuse concentric coronary distribution of CAV, which may not be angiographically apparent until CAV has significantly progressed [21]; moreover, it does allow only a macroscopic evaluation of ventricular function and does not offer assessment of microvascular CAV disease, which is independently prognostic in OHT recipients [15, 22]. Intravascular ultrasound (IVUS) offers greater sensitivity for CAV detection [23, 24], however, IVUS is also an invasive procedure associated with major risks from left heart catheterization and is limited to epicardial coronary artery assessment. In these regards, there is scope for CMR to play a role in detection of CAV as a non-invasive diagnostic tool, due to its capacity to assessing global and focal myocardial structural and functional anomalies and perfusion deficits due to epicardial or microvascular disease, when rest/stress perfusion sequences are acquired as well.

Early CAV diagnosis is essential as the occurrence of allograft dysfunction will negatively affect the prognosis, with lower survival than in patients than without allograft dysfunction [25–27]. CMR has the potential to provide more in-depth characterization of LV myocardial mechanics with FTS, and myocardial tissue characterization (as well as epicardial or microvascular related perfusion deficits, when rest/stress perfusion sequences are added) prior to the onset of clinically significant allograft dysfunction, at both a regional and global level and to assist in identify these patients at an earlier stage. The benefits of FTS include that it can be applied as a post-processing step to routine cine images, with no additional sequence acquisitions required, and offers improved acquisition windows when compared to speckle tracking echocardiography.

*Study Limitations*: Study limitations include that this is a small sample size, single-center study, as is seen with many OHT studies. Larger, multi-center studies are required to validate our findings. No segmental analysis of T1, T2, ECV and strain was performed, only global LV parameters. Future studies should investigate the impact of CAV on regional changes in tissue and function. Given the nature of this study’s design, CAV grade was determined from clinically requested ICA, RHC and TTE data, and no advanced intracoronary imaging (IVUS or optical coherence tomography) data was consistently available for each participant to include in our analysis. Furthermore, there were no invasive measures of microvascular dysfunction available for the selected patients, such as microvascular resistance measures or coronary flow reserve, to compare with our findings. Therefore, the description of disease progression was limited to epicardial coronary disease. Qualitative CMR measures such as late gadolinium enhancement or stress perfusion were not included in this analysis; however, stress perfusion may also supplement detection of CAV [16, 17]. In order to evaluate patients with mild-moderate CAV that can be detected with ICA, patients who received CMR 0–6 years post-OHT were included in this study, as this timeframe is comparable with the reported time frame by which 40–50% of CAV cases are angiographically apparent, most of which are mild-moderate CAV [28]. Further studies are required to assess more severe CAV grades and patient’s with OHT irrespective of the timeframe post-CMR.

## Conclusion

In conclusion, multiparametric quantitative cardiac tissue and functional MRI parameters have the potential for non-invasive detection of mild to moderate CAV in OHT recipients. Correlations between altered tissue characteristics and strain rates are suggestive of a relationship between myocardial structural alterations and subclinical dysfunction in CAV.

## Data Availability

Data reported in the manuscript, part of an ongoing study at Northwestern University, Department of Radiology, can be accessed by contacting the corresponding author (S.Q.)

## Abbreviations

2D: Two-dimensional
ACAR: Acute cardiac allograft rejection
AUC: Area under the curve
bSSFP: Balanced steady state free precession
CAV: Coronary allograft vasculopathy
CI: Confidence interval
CMR: Cardiac magnetic resonance imaging
ECV: Extracellular volume fraction
FTS: Feature tracking strain
GRAPPA: Generalized autocalibrating partially parallel acquisition
ICA: Invasive coronary angiography
ISHLT: International Society of Heart and Lung Transplantation
IVUS: Intravascular ultrasound
LV: left Ventricle/left ventricular
MOLLI: Modified Look Locker Inversion recovery
OHT: Orthotopic heart transplant
RHC: Right heart catheterization
ROC: Receiver operator characteristics
RV: Right ventricle/right ventricular
TE: Echo time
TR: Repetition time
TTE: Transthoracic echocardiography
